# Prognostic significance of PET/CT false-positive cervical lymph nodes in head and neck cancer

**DOI:** 10.1038/s41598-026-51823-1

**Published:** 2026-05-08

**Authors:** Alisa Mohebbi, Afshin Mohammadi

**Affiliations:** 1 Tehran University of Medical Sciences, Tehran, Iran; 2 Urmia University of Medical Sciences, Urmia, Iran

**Keywords:** Head and neck squamous cell carcinoma (HNC), Lymph node metastasis (LNM), Prognosis, PET/CT, Cancer, Oncology

## Abstract

Cervical lymph node metastasis assessment determines treatment strategy and prognosis in head and neck squamous cell carcinoma (HNC). PET/CT is the standard staging modality but suffers from high false-positive (FP) rates with a substantial portion of imaging-suspicious nodes proving pathologically negative. A fundamental knowledge gap persists; whether imaging positivity itself, despite ultimate pathologic negativity, provides prognostic significance sufficient to justify treatment escalation. This study directly answers this critical clinical question by comparing prognostic outcomes between patients with imaging-positive-but-pathology-negative nodes (FP) and those with concordantly imaging-negative nodes (TN), determining whether the additional imaging positivity differential translates into measurable survival or recurrence disparities. A total of 176 newly diagnosed HNC patients from the ACRIN 6685 cohort undergoing PET/CT staging with complete pathologic confirmation were included. Patients were stratified into FP and TN groups. Cox and competing-risk regression analyses compared recurrence-free survival, overall survival, and HNC-specific survival between two cohorts, adjusted for potential prognostic confounders. Among 176 patients, 137 (77.8%) demonstrated FP findings on PET. Imaging positivity provided no prognostic value; FP and TN cohorts showed equivalent overall survival and HNSCC-specific mortality. Quantitative PET nodal burden (number of suspicious lymph node levels) minimal recurrence association (*p* = 0.013), but binary nodal presence did not. Imaging positivity alone, when pathology is negative, does not predict worse outcomes. This imaging-pathology discordance carries no prognostic penalty, allowing clinicians to base treatment decisions and prognostic counseling on surgical pathology results rather than escalating care based solely on imaging appearance.

​.

## Introduction

Accurate assessment of cervical lymph node metastasis (LNM) in head and neck squamous cell carcinoma (HNC) is fundamental to therapeutic strategy, prognostic stratification, and long-term survival outcomes^1–4^. Positron emission tomography/computed tomography (PET/CT) has emerged as the standard imaging modality for HNC lymph node staging, providing complementary metabolic and anatomic information unavailable from either modality alone^5,6^. Despite its widespread clinical use, PET/CT is limited by an important drawback: its positive predictive value is often inadequate. In a recent systematic review and meta-analysis, 8 of 16 included studies reported a positive predictive value of ≤ 60%, indicating a correspondingly high prevalence of false-positive (FP) findings^7^.

False-positive findings—defined as imaging-positive but pathology-negative cervical LNM—pose a substantial clinical challenge that extends beyond diagnostic accuracy metrics. In contrast, true-negative (TN) patients show concordantly negative imaging and pathology findings. Accordingly, a fundamental knowledge gap remains: whether imaging positivity despite pathologic negativity confers additional prognostic significance. In addition, radiologists’ diagnostic certainty differs substantially between these groups: TN cases benefit from imaging-pathology concordance and greater confidence in benignity, whereas FP cases create discordance and diagnostic uncertainty that may influence clinical decision-making and subsequent patient management. Although several investigations have quantified the prevalence of FP cervical LNM findings on PET/CT and highlighted their implications for staging accuracy and clinical decision-making, there remains a conspicuous absence of data assessing whether such imaging-pathology discordance per se imparts any measurable differences in overall survival or recurrence-free survival.

To address this critical knowledge gap, the present prospective multicenter study compares FP and TN LNM populations in HNC, a distinction that has been underrepresented in the literature. By directly comparing disease recurrence, overall survival, and HNC-specific survival between FP and TN cohorts, this study evaluates whether imaging positivity in FP patients constitutes a prognostic risk factor that warrants changes in management or whether such findings are benign incidental observations that merit clinical reassurance.

## Materials & methods

This post-hoc study was done on the American College of Radiology Imaging Network (ACRIN) 6685 prospective multicenter cohort^8,9^. The primary study involved 24 centers in the United States. Data collection was conducted with institutional review board approval from all participating institutions, and all participants provided written informed consent. Since this study used fully de-identified data, the local institutional review board of Urmia University of Medical Sciences exempted it from further approval or consent requirements. All research was conducted in accordance with relevant human ethical guidelines and regulations.

### Study population and eligibility criteria

Initial inclusion criteria for the parent trial were as follows: (1) age ≥ 18 years and willingness to provide informed consent; (2) PET/CT imaging within 14 days before planned surgical resection to ensure close temporal proximity between imaging and pathologic confirmation and to minimize interval disease progression that could compromise imaging-pathology correlation; (3) newly diagnosed, pathologically confirmed HNC; and (4) at least one neck side classified as cN0 and considered a candidate for neck dissection. Initial exclusion criteria for the original trial were: (1) distant metastatic disease, defined as pathologically or radiologically confirmed metastases beyond the cervical lymph nodes and primary tumor site; (2) prior chemotherapy, radiotherapy, or surgical resection in the head and neck region thereby excluding all patients with recurrent or second primary malignancies; (3) claustrophobia or any other contraindication to FDG-PET/CT imaging; and (4) nasopharyngeal, cutaneous, thyroid, sinonasal, or salivary gland malignancies.

The parent trial enrolled a total of 292 participants across all participating institutions, meeting all original inclusion and exclusion criteria specified above. For the current study, additional exclusions were applied to ensure data completeness and analytical coherence appropriate for the specific research question: (1) 46 patients were excluded due to absent follow-up status data or absent pathology reporting from the surgical specimen. The requirement for both completed surgical pathology and documented follow-up ensured that all remaining participants had an established reference standard for cervical lymph node status and documented prognostic clinical outcomes; (2) Additional 67 patients with pathologically confirmed positive cervical LNM (defined as the presence of at least one malignant lymph node on histopathologic examination of the surgical specimen) were excluded, as the current investigation specifically addresses the dichotomy between FP and TN patients; and (3) 3 participants with missing critical demographic or imaging variables due to missing critical demographic or imaging variables necessary for clinical regression-based adjustments. The sequential exclusion process yielded a final cohort of 176 patients.

### PET/CT imaging acquisition and interpretation

All study participants underwent FDG-PET/CT examination within 14 days prior to planned surgical resection. The FDG-PET/CT examinations were conducted according to standardized multicenter protocols, with identical acquisition parameters across all participating institutions to ensure technical consistency and comparability of findings. Prior to imaging, study participants fasted for a minimum of 4 h. Blood glucose levels were verified to be < 200 mg/dL immediately before FDG injection. FDG dosage adjusted for body weight with standard activity of 10–20 millicuries. Integrated FDG-PET/CT imaging was performed on multidetector hybrid scanners available at participating institutions. The CT acquisition component was performed without intravenous contrast media using standard parameters (120 kVp, 90–150 mAs, 2.5 mm slice thickness).

Consistent with real-world clinical workflow, lymph node positivity on PET/CT was determined by readers using a holistic visual assessment of uptake intensity relative to background, nodal morphology, and anatomic context. We did not apply a predefined maximum standardized uptake value (SUVmax) threshold because such values are susceptible to inter-scanner variability and are not routinely used as the sole criterion for nodal malignancy in contemporary HNC practice. PET/CT examinations were interpreted by at least two board-certified nuclear medicine physicians or radiologists with subspecialty expertise in oncologic PET imaging at each participating institution. The assessments occurred blinded, with each reader providing diagnostic impressions regarding nodal status without knowledge of the other readers’ interpretation. After completion of the independent assessments, the interpretations were reviewed, concordant and discordant findings were identified, and a final diagnostic determination was established collaboratively for each case. The interpretation protocol specifically mandated separate, modality-focused assessment of PET and CT components to optimize the diagnostic contribution of each imaging technique and prevent inappropriate over-reliance on a single modality. Importantly, the interpreting physicians were blinded to surgical pathology results.

Cervical lymph nodes at all nodal levels (Level IA through VI) were systematically characterized through a comprehensive approach integrating visual assessment of metabolic activity and anatomic features. The assessment encompassed both visual inspection and quantitative measurement of FDG uptake on the PET component, combined with anatomic evaluation on the CT component. For standardization and reproducibility across all study sites, visual positive nodal FDG uptake for PET component was defined suspicious for LNM while lymph nodes with suspicious CT findings (e.g., short-axis diameter ≥ 1.0 centimeter, round or oval morphology, central necrosis, heterogeneous internal density) were considered malignant for CT component. To standardize diagnostic categorization and enable communication of readers certainty regarding cervical lymph node status, a five-point ordinal scale was employed; category 1 (definitely benign), category 2 (probably benign), category 3 (indeterminate), category 4 (probably malignant), and category 5 (definitely malignant). Nodes classified as categories 3–5 were recorded as metastatic. Nodes classified as categories 1–2 were recorded as negative/benign.

Within this framework of comprehensive nodal assessment, definitions of FP and TN were established based on imaging-pathology concordance patterns; FP findings were defined as study participant harboring at least one cervical lymph node demonstrating probability of malignancy on imaging modality (corresponding to categories 3–5) that subsequently proved entirely negative for metastatic disease on pathologic examination of the surgical specimen. TN findings were defined as study participants demonstrating no suspicious lymph nodes on imaging (corresponding to categories 1–2) and subsequently had no pathologic evidence of cervical lymph node malignancy on examination of surgical specimens.

### Treatment protocol

All study participants underwent surgical resection of the primary tumor with concurrent cervical neck dissection. The specific type of neck dissection including radical neck dissection, modified radical neck dissection, or selective neck dissection targeting specific nodal levels was determined by the treating surgeon based on primary tumor location, size, grade, and surgeon judgment regarding the extent of nodal disease and necessity for comprehensive lymphadenectomy. Following surgical resection, adjuvant systemic therapy (chemotherapy and/or radiotherapy) was administered according to institutional protocols and treating oncologist assessment of disease risk and benefit-risk balance for additional treatment. The decision to recommend adjuvant therapy was influenced by multiple factors including primary tumor stage, grade, etc. on imaging, or at surgery, presence of adverse pathologic features (perineural invasion, lymphovascular invasion), and patient tolerance for additional treatment.

### Patient follow-up and outcome assessment

The primary clinical outcomes examined in this prognostic comparative analysis were: (1) recurrence-free survival, defined as time from surgery to first documented local, regional, or distant recurrence confirmed by imaging, clinical examination, or histopathology; (2) overall survival defined as time from surgery to death from any cause; and (3) HNC-specific survival, defined as time from surgery to death attributable to HNC. Local recurrence was defined as tumor recurrence at or immediately adjacent to the primary tumor site within the head and neck region, regional recurrence as recurrence within the cervical lymphatic basins, and distant recurrence as metastatic disease at non–head and neck sites (e.g., lung, liver, bone, or other distant organs). For the purposes of time-to-event analysis, these patterns were combined into a single composite endpoint of recurrence, and recurrence-free survival was calculated from enrollment to the first occurrence of local, regional, or distant recurrence.

### Statistical analysis

All statistical analyses were performed using Stata version 17.0 and Medcalc version 23.2. The analytical strategy was specifically designed to conduct head-to-head prognostic comparison of two distinct patient populations (TN vs. FP) across three clinically meaningful survival endpoints encompassing HNC-specific mortality, disease recurrence, and all-cause mortality. The competing risk regression analysis employing the Fine-Gray method was implemented for analysis of the HNC-specific mortality endpoint while for other two endpoints Cox regression analysis was implemented. For all three endpoints, both univariable and multivariable regression models were constructed. Univariable analyses examined the association between TN versus FP without adjustment for other variables. In multivariable Cox proportional hazards and competing risk models, we adjusted for age at enrollment, sex, and TNM stage classification, selected a priori based on their established prognostic relevance in HNC. For all models, hazard ratios (HRs) were calculated and reported with corresponding 95% confidence intervals (CIs). All statistical tests were two-tailed, with statistical significance defined a priori as *p* < 0.05. In addition to regression model-based analyses, Kaplan-Meier survival curves were constructed for endpoints.

## Results

### Patient characteristics

The study cohort comprised 176 patients with newly diagnosed HNC who underwent surgical staging with pathologic confirmation of cervical lymph node status. The median follow-up duration was 944.8 days (95% CI: 878.5–1011.1 days). The population was predominantly male, with 113 patients (64.2%) being men. The mean age at enrollment was 60.4 years (95% CI, 58.8–62.0 years). 27 patients (15.3%) received adjuvant chemotherapy and 73 patients (41.5%) received adjuvant radiotherapy after surgery. Regarding imaging-pathology concordance patterns on PET/CT, 137 patients (77.8%) were classified as FP on PET imaging. On CT imaging, 41 patients (23.3%) were classified as FP. The remaining patients in both modalities (39 on PET and 135 on CT) were classified as TN, demonstrating concordant imaging-pathology findings with no suspicion for LNM. Detailed patient characteristics are provided in Table 1. All baseline clinical and demographic variables, including primary tumor site and T-stage, showed p values > 0.05 when comparing FP and TN groups for both PET- and CT-based nodal assessment.

**Table 1:** Baseline characteristics of study population.

Characteristic	Value
Male, n (%)	113 (64.2%)
Mean Age, years (95% CI)	60.4 (58.8–62.0)
Initial Clinical TNM Stage I II III IV	2 (1.1%) 101 (57.4%) 31 (17.6%) 42 (23.8%)
Initial Clinical Tumor Stage (7th AJCC TNM) T1, n (%) T2, n (%) T3, n (%) T4, n (%)	2 (1.1%) 105 (59.6%) 33 (18.7%) 36 (20.4%)
Initial Clinical Nodal Stage (7th AJCC TNM) N0, n (%) N1, n (%) N2A, n (%) N2B, n (%) NX, n (%)	158 (89.7%) 10 (5.6%) 2 (1.1%) 5 (2.8%) 1 (0.5%)
Recurrence Events, n (%)	32 (17.0%)
Total Deaths, n HNC-Related Deaths, n (%) Non-HNC Deaths, n (%)	30 20 (66.7%) 10 (33.3%)
Primary Tumor Site Tongue Floor of Mouth Larynx Alveolar Ridge Tonsil Retromolar Trigone Buccal Mucosa Hypopharynx Hard Palate Other	55 (31.2%) 32 (18.1%) 30 (17.0%) 17 (9.6%) 13 (7.3%) 11 (6.2%) 7 (3.9%) 4 (2.2%) 2 (1.1%) 5 (2.8%)
FP on PET, n PET FP with 1 LNL involvement, n (%) PET FP with 2 LNLs involvement, n (%) PET FP with 3 LNLs involvement, n (%) PET FP with 4 LNLs involvement, n (%) PET FP with ≥ 5 LNLs involvement, n (%)	137 47 (34.3%) 41 (29.9%) 22 (16.0%) 12 (8.7%) 15 (10.9%)
FP on CT, n CT FP with 1 LNL involvement, n (%) CT FP with 2 LNLs involvement, n (%) CT FP with 3 LNLs involvement, n (%) CT FP with 4 LNLs involvement, n (%)	41 24 (58.5%) 9 (21.9%) 4 (9.7%) 4 (9.7%)

### PET-based nodal assessment and survival outcomes

**Fig. 1:**
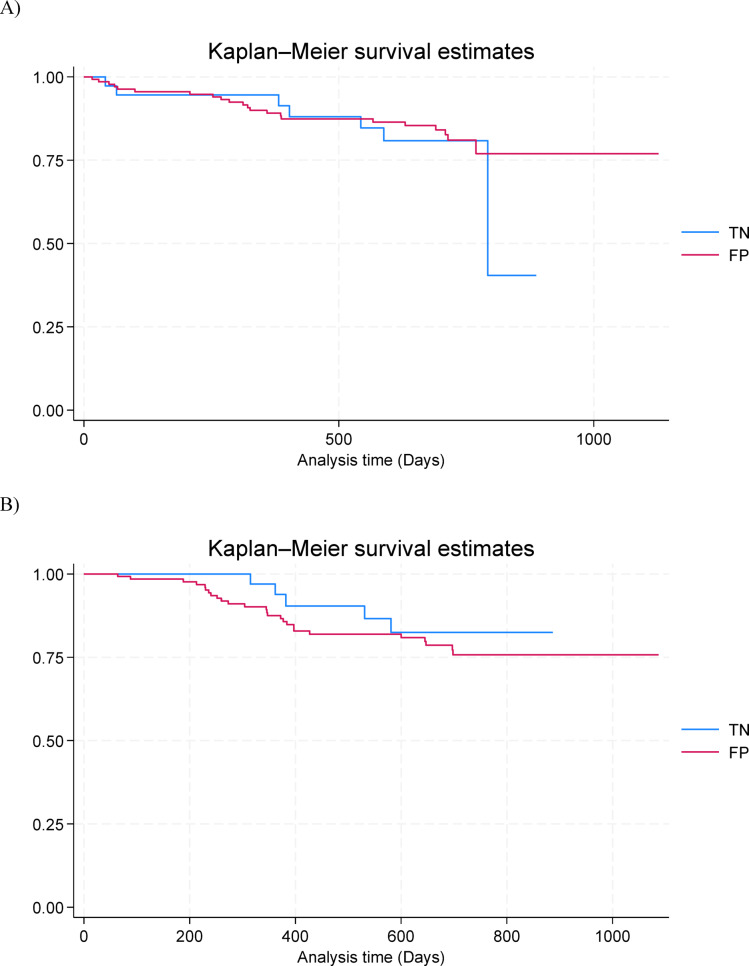

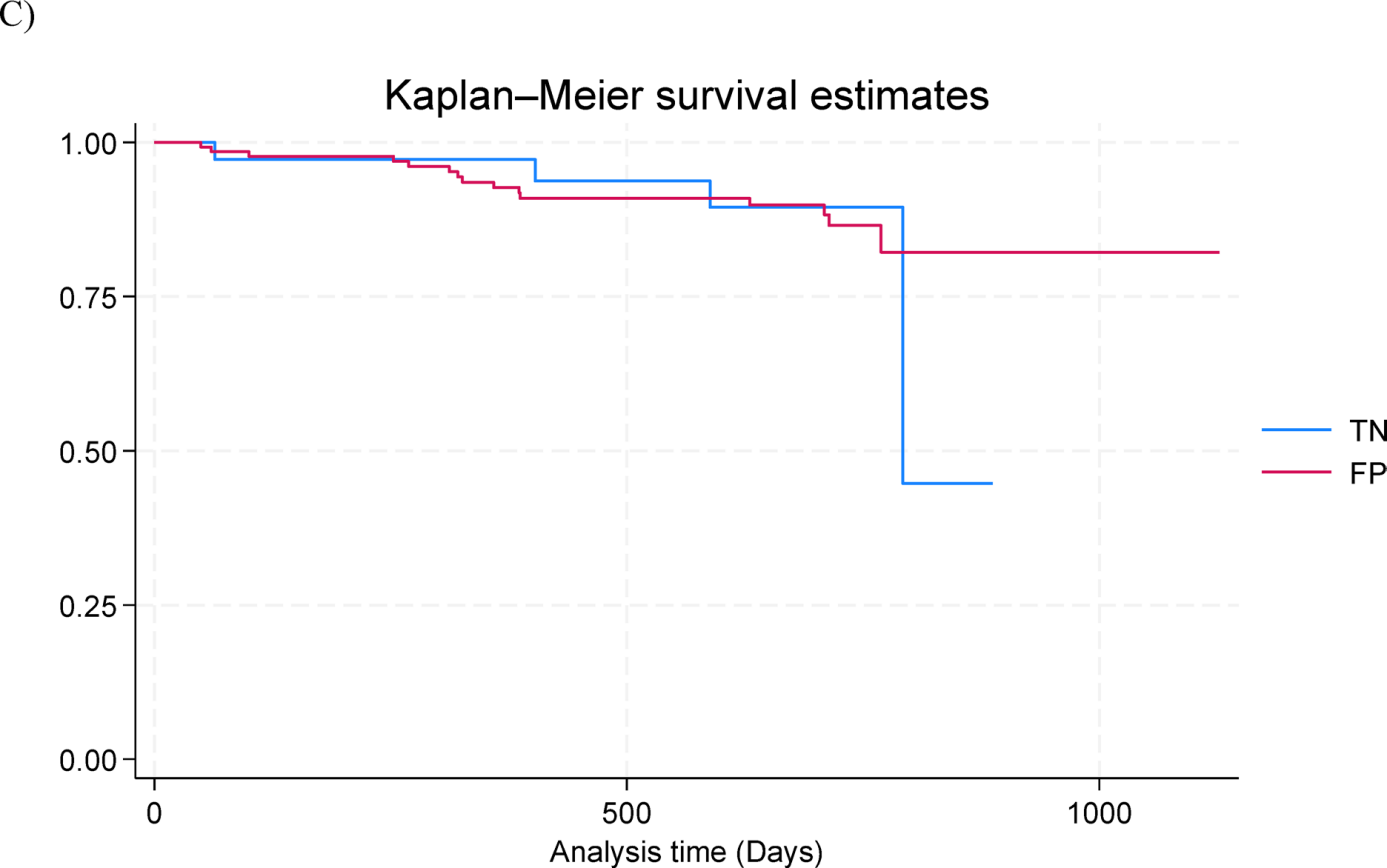
Kaplan–Meier curves for overall survival (**A**), recurrence-free survival (**B**), and HNC-specific survival (**C**) by PET imaging status (FP vs. TN).

PET imaging showed similar prognostic implications between the FP and TN cohorts across the three survival endpoints examined. Comparative analysis showed that PET-detected suspicious lymph nodes, despite subsequent pathologic negativity, conferred no additional prognostic risk for overall survival or HNC-specific survival relative to pathologically concordant negative cases, challenging the assumption that imaging positivity warrants intensified surveillance or therapeutic modification. Notably, quantitative assessment of the number of suspicious nodal levels showed a small but statistically significant association with recurrence risk in both unadjusted and adjusted analyses (*p* = 0.026 and *p* = 0.013, respectively), suggesting that imaging-detected nodal burden may carry limited predictive value for recurrence despite pathologic negativity. By contrast, binary nodal presence was not statistically significant for any survival endpoint, suggesting that quantitative grading provides slightly better prognostic discrimination than dichotomous classification. The PET analysis results are presented in Table 2. Kaplan–Meier survival curves comparing FP and TN groups for overall survival, recurrence-free survival, and HNC-specific survival are presented in Fig. 1.

**Table 2:** Prognostic Outcomes by PET-Based Nodal Assessment.

Outcome	Analysis Type	HR (95% CI) – Unadjusted	*p*-value – Unadjusted	HR (95% CI) – Adjusted	*p*-value – Adjusted	Mean Survival (95% CI), days
Overall Survival	Quantitative	0.92 (0.74–1.16)	0.511	0.92 (0.72–1.18)	0.543	—
	Binary	0.82 (0.35–1.92)	0.649	0.68 (0.28–1.68)	0.411	FP: 693 (660–726) TN: 691 (625–756)
Recurrence-Free Survival	Quantitative	1.19 (1.02–1.40)	0.026	1.24 (1.04–1.48)	0.013	—
	Binary	1.43 (0.55–3.75)	0.457	1.50 (0.56–3.99)	0.409	FP: 528 (506–550) TN: 557 (521–593)
HNC-Specific Survival	Quantitative	0.95 (0.74–1.21)	0.681	0.95 (0.72–1.25)	0.732	—
	Binary	0.99 (0.34–2.88)	0.986	0.85 (0.27–2.63)	0.788	FP: 719 (692–746) TN:728 (681–776)

### CT-based nodal assessment and survival outcomes

**Fig. 2:**
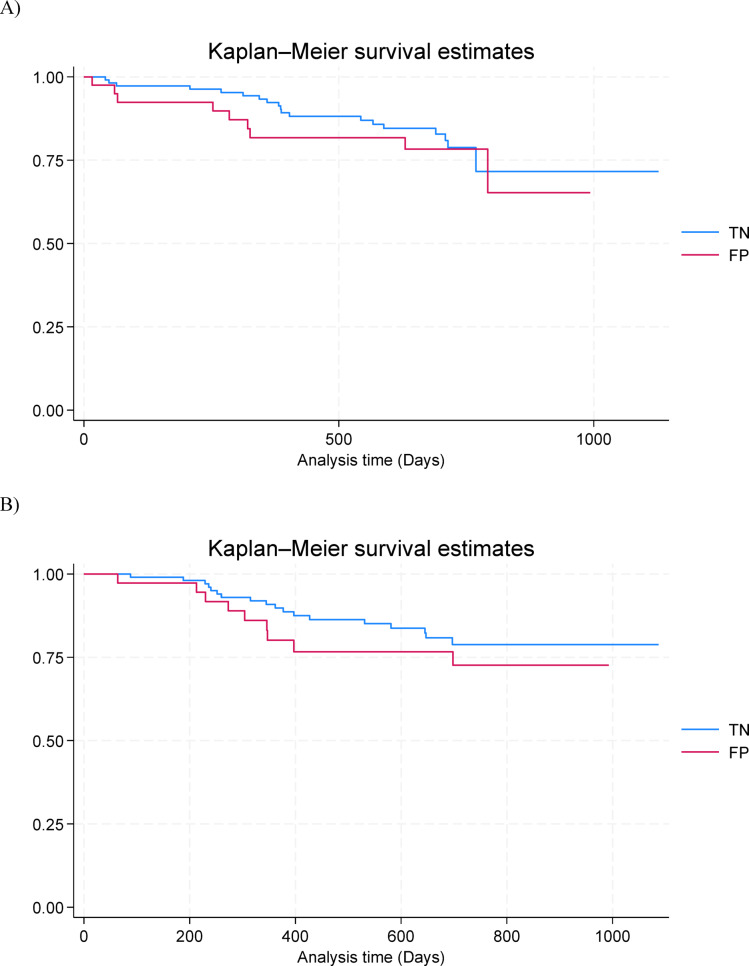

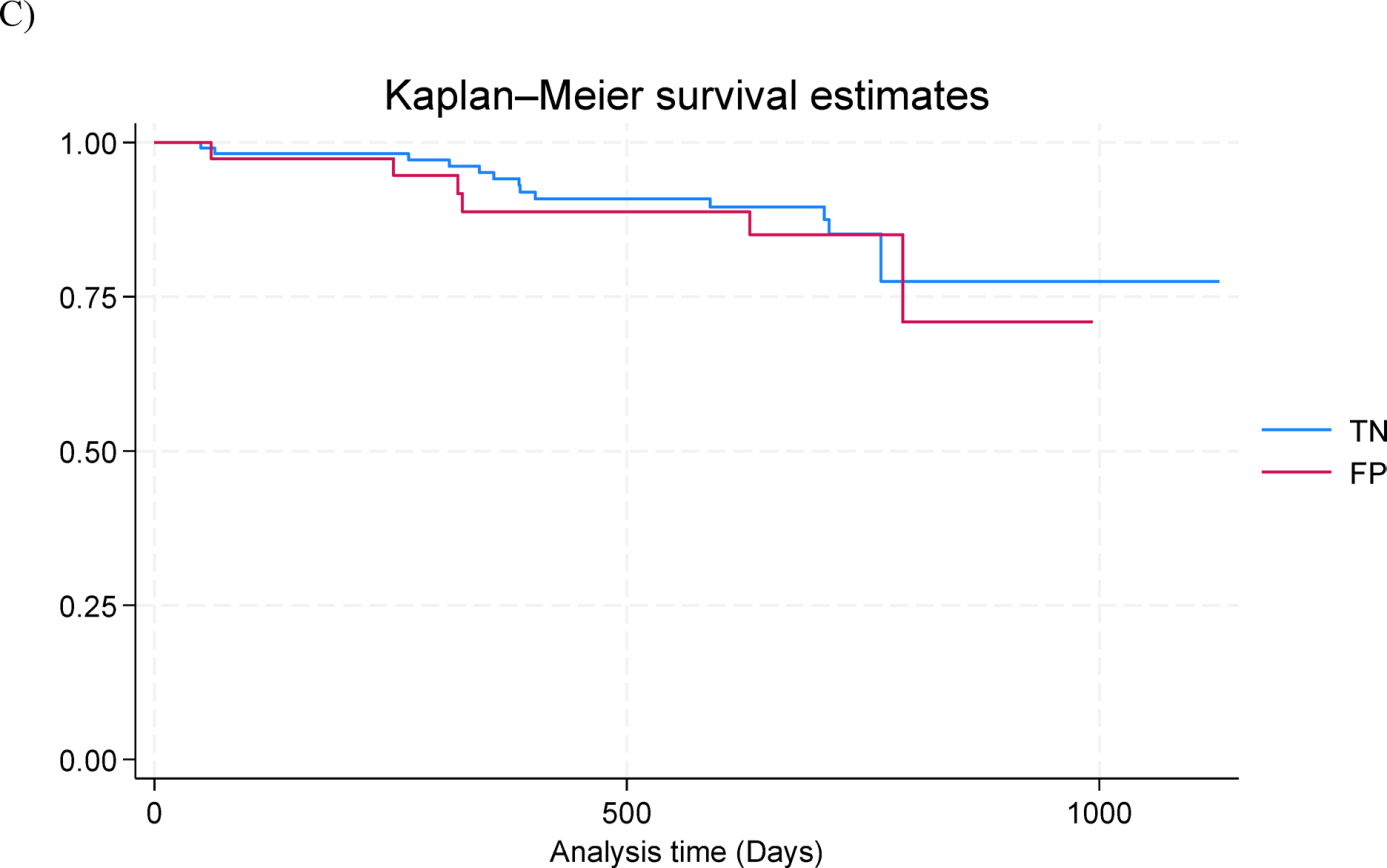
Kaplan–Meier curves for overall survival (**A**), recurrence-free survival (**B**), and HNC-specific survival (**C**) by CT imaging status (FP vs. TN).

CT assessment of cervical lymph nodes demonstrated no prognostic differentiation between FP and TN patient populations across endpoints across both unadjusted and adjusted analyses. Neither the binary presence or absence of suspicious lymph nodes nor the numerical measure of suspicious nodal levels conferred significant prognostic value in any analysis paradigm. The absence of prognostic benefit extended uniformly across all three examined survival metrics. Mean survival estimates demonstrated numerical overlap between groups with overlapping confidence intervals, while hazard ratios approximated unity across all endpoints, reflecting no differential risk stratification (Table 3). Kaplan–Meier survival curves comparing FP and TN groups for overall survival, recurrence-free survival, and HNC-specific survival are presented in Fig. 2.

Analysis of modality-specific discordance revealed distinct patterns of FP findings. Among 151 cases with complete imaging data, 26 (17.2%) demonstrated TN across both modalities and 37 (24.5%) were FP on both PET and CT. On the other hand, 4 (2.6%) were FP on CT alone but TN on PET. Notably, 84 cases (55.6%) exhibited PET FP despite CT TN. This distribution indicates that most FP findings were PET-specific, potentially reflecting the modality’s heightened sensitivity to metabolic activity that may not correlate with malignant pathology.

**Table 3:** Prognostic Outcomes by CT-Based Nodal Assessment.

Outcome	Analysis Type	HR (95% CI) – Unadjusted	*p*-value – Unadjusted	HR (95% CI) – Adjusted	*p*-value – Adjusted	Mean Survival (95% CI), days
Overall Survival	Quantitative	1.07 (0.73–1.56)	0.699	0.99 (0.68–1.45)	0.991	—
	Binary	1.24 (0.55–2.77)	0.593	1.25 (0.53–2.97)	0.603	FP: 658 (579–738) TN: 699 (665–732)
Recurrence-Free Survival	Quantitative	1.17 (0.82–1.67)	0.363	1.15 (0.79–1.68)	0.452	—
	Binary	1.45 (0.65–3.24)	0.356	1.43 (0.62–3.33)	0.396	FP: 510 (459–560) TN: 540 (519–562)
HNC-Specific Survival	Quantitative	0.90 (0.59–1.38)	0.640	0.85 (0.54–1.35)	0.512	—
	Binary	1.13 (0.42–3.02)	0.802	1.20 (0.37–3.82)	0.754	FP: 704 (638–771) TN: 720 (691–749)

## Discussion

This prospective multicenter study addresses a major gap in HNC prognostic knowledge by examining whether PET or CT positivity in the setting of pathologic negativity provides prognostic information sufficient to alter clinical management. The findings demonstrate that neither PET- nor CT-detected suspicious cervical lymph nodes, when pathologically negative, predict differences in survival or recurrence outcomes compared with concordantly negative imaging–pathology findings. This observation has relevant clinical implications, as it provides evidence that may help refine the interpretive framework for FP lymph node findings in contemporary HNC imaging practice and reduce a persistent source of diagnostic uncertainty that influences clinical decision-making in routine patient care. Historically, the interpretation of imaging-positive lymph nodes has operated under a presumptive model in which imaging positivity, even in isolation, creates psychological and clinical pressure toward more aggressive intervention whether through expansion of neck dissection templates, intensification of adjuvant therapy, or closer surveillance protocols. The present study directly challenges this assumption, demonstrating that the imaging-pathology discordance itself does not translate into measurable prognostic parameter when pathologic examination confirms the absence of metastatic disease.

The absence of prognostic differentiation between FP and TN cohorts for the primary endpoints of overall survival and HNC-specific mortality constitutes powerful evidence that pathologic confirmation of nodal negativity fully captures the true biologic disease status, at least insofar as it pertains to survival outcomes. When pathologic examination of the surgical specimen definitively establishes the absence of metastatic disease in lymph nodes that appeared suspicious on imaging, the patient’s prognostic status follows that of other pathologically node-negative patients, regardless of the preceding imaging appearance. This observation validates the complete dominance of surgical pathology as the reference standard and reinforces the principle that diagnostic test performance must ultimately be anchored to clinically meaningful outcomes rather than to abstract measures of concordance or discordance. The implications are substantial; a clinician encountering an FP finding need not escalate diagnostic uncertainty into modifying the surgical or adjuvant treatment plan based solely on the imaging appearance when pathologic confirmation clarifies the true nodal status.

However, the numerical assessment of suspicious nodal levels on PET imaging demonstrated statistically significant association with recurrence risk in both unadjusted and adjusted analyses, requiring careful interpretation regarding the clinical utility of this finding. The observation that the number of PET-suspicious nodal levels, but not their binary presence, achieved statistical significance suggests a nuanced relationship between imaging-detected nodal burden and recurrence risk. This distinction between quantitative and categorical assessment carries important mechanistic implications; perhaps the imaging-detected extent of metabolic nodal activity reflects a more diffuse pattern of systemic disease or increased primary tumor aggressiveness rather than occult cervical nodal metastases per se. Alternatively, the number of suspicious nodal levels might serve as a proxy marker for tumor biology or host immune response that influences recurrence propensity independently of lymph node involvement status. However, we explicitly acknowledge that these mechanistic interpretations remain speculative and lack direct biological validation in the current study. They represent testable hypotheses requiring confirmation through future molecular investigations.

These findings have direct implications for multidisciplinary tumor board workflows. When encountering PET/CT-positive nodes without pathological confirmation, our results suggest treatment escalation is unwarranted. However, documentation of suspicious nodal levels remains valuable for surveillance planning. Tumor boards should: (1) prioritize pathological findings over isolated imaging results in treatment decisions; (2) maintain detailed mapping of PET-positive nodal levels for recurrence monitoring; and (3) recognize that metabolic activity without pathological confirmation may represent inflammatory processes rather than metastatic disease. This approach balances the diagnostic value of advanced imaging with the prognostic primacy of pathological confirmation.

An additional consideration is that some lymph nodes classified as FP may, in fact, harbor micrometastatic disease that was not detectable by histopathological assessment. It is therefore possible that a subset of nodes deemed pathologically negative contained occult micrometastases or isolated tumor cells below the detection threshold. Such misclassification would tend to blur the distinction between FP and TN nodal status and may partly account for any residual adverse prognostic signal associated with the FP group. Future studies incorporating more intensive histopathologic evaluation or molecular techniques could help clarify the extent to which imaging positivity reflects genuinely FP findings versus detection of subclinical nodal tumor burden.

An interesting observation of our study is that the numerical assessment of suspicious nodal levels on PET imaging showed a statistically significant but minimal association with the risk of recurrence in both unadjusted and adjusted analyses. From a clinical perspective, the effect size observed in our cohort does not justify altering the standard indications for neck dissection or adjuvant treatment solely on the basis of the number of PET-positive nodal levels. However, this parameter may have value as an adjunctive risk marker that could refine postoperative risk stratification when considered alongside established prognostic factors such as tumor stage, margin status, and extracapsular extension. In particular, patients with a higher number of suspicious nodal levels on PET, despite negative pathological nodal findings, might be candidates for closer surveillance or for inclusion in future trials evaluating risk-adapted adjuvant strategies. Given the limited magnitude of the effect and the observational design, these findings should be viewed as hypothesis-generating and warrant validation in larger, independent cohorts before they can be translated into actionable clinical algorithms.

The landscape of HNC lymph node assessment stands at a point where emerging methodologies and refined understanding of imaging-pathology discordance create opportunities for advancement. Future investigations should pursue radiomics-based approaches that move beyond traditional visual assessment to extract quantitative imaging biomarkers from PET and CT data, potentially identifying computational signatures within FP findings that predict recurrence risk or treatment response with precision unavailable through conventional radiologic interpretation^10–12^. Additionally, prospective studies incorporating molecular and genomic profiling of the primary tumor may be prioritized to investigate whether imaging-detected but pathologically-negative nodal regions reflect circulating tumor DNA, disseminated tumor cells, or altered lymphatic drainage that presages systemic micrometastatic disease detectable only through advanced molecular methods^13^. Furthermore, investigation of dynamic contrast-enhanced MRI and diffusion-weighted imaging as complementary or alternative modalities for cervical lymph node assessment is essential, as these techniques provide distinct parameters that might elaborate on the mechanistic basis for imaging-pathology discordance^14–18^.

This investigation, while prospectively designed and rigorously executed across multiple institutions, operates within several important constraints that warrant explicit acknowledgment and careful consideration when applying findings to diverse clinical contexts. First, the study population comprised predominantly male patients (64.2%) with mean age of 60.4 years, potentially limiting the generalizability of findings to younger patients, female-predominant HNC subtypes, or specific demographic populations that may demonstrate different imaging characteristics or prognostic trajectories. Second, our study exclusively included cN0 patients managed with upfront surgery and elective neck dissection. This design allowed for detailed histopathologic correlation but inherently restricts the generalizability of our findings to surgically treated cN0 populations, inherently restricting applicability to non-surgical patient populations treated with primary chemoradiation therapy, immunotherapy, or other organ-preservation strategies where imaging-pathology concordance patterns cannot be established. Third, the study integration did not characterize the benign etiologies underlying the FP findings, such as reactive lymphadenopathy from infection, inflammatory processes, prior chemotherapy effects, or radiation-related changes, understanding the mechanistic basis for FP findings. Furthermore, our analysis is constrained by incomplete data on several important prognostic factors. In particular, HPV status, detailed smoking history, and alcohol consumption were not systematically available for all patients and therefore could not be incorporated into our multivariable models. Given the well-established influence of these variables on outcomes in head and neck squamous cell carcinoma, the possibility of residual confounding cannot be excluded, even after adjustment for the clinicopathologic factors included in our models. Additionally, the cohort is heavily weighted toward oral cavity cancers. This distribution differs from the overall epidemiological profile of HNC seen in general clinical practice and primarily reflects the referral patterns and case mix of the participating tertiary care centers, where surgically managed oral cavity malignancies are disproportionately represented. Consequently, the generalizability of our findings to the broader head and neck cancer population is limited. Finally, an additional limitation is the heterogeneity of primary tumor sites within the cohort, which included oral cavity, oropharyngeal, laryngeal, and hypopharyngeal squamous cell carcinomas. Given the sample size and number of outcome events, we were not adequately powered to conduct robust, site-specific multivariable analyses, and the present findings should therefore be interpreted as pertaining to surgically managed upper aerodigestive tract squamous cell carcinomas as a group.

## Conclusion

This prospective multicenter study demonstrated that cervical lymph node FP findings on PET/CT imaging do not predict differential overall survival or HNC-specific mortality compared to TN concordant findings when pathology confirms nodal negativity. These results provide robust evidence that imaging-pathology discordance, while diagnostically imperfect, does not translate into worse clinical outcomes. Clinicians can confidently base treatment decisions and prognostic counseling on surgical pathology findings without therapeutic escalation based solely on preceding imaging appearance, thereby simplifying clinical decision-making and reducing unnecessary treatment intensification in this patient population.

## Data Availability

The data that support the findings of this study are available from ACRIN 6685 trial through the Cancer Data Access System (CDAS) at The Cancer Imaging Archive (TCIA), hosted by the National Cancer Institute (NCI) but restrictions apply to the availability of these data, which were used under license for the current study, and so are not publicly available. Data are however available from the authors upon reasonable request and with permission of TCIA.
